# Remote Virtual Interactive Agents for Older Adults: Exploring Its Science via Network Analysis and Systematic Review

**DOI:** 10.3390/healthcare13172253

**Published:** 2025-09-08

**Authors:** Michael Joseph Dino, Chloe Margalaux Villafuerte, Veronica A. Decker, Janet Lopez, Luis Ezra D. Cruz, Gerald C. Dino, Jenica Ana Rivero, Patrick Tracy Balbin, Eloisa Mallo, Cheryl Briggs, Ladda Thiamwong, Mona Shattell

**Affiliations:** 1 College of Nursing, University of Central Florida, 6825 Lake Nona Blvd, Orlando, FL 32827, USA; veronica.decker@ucf.edu (V.A.D.); janet.lopez@ucf.edu (J.L.); ladda.thiamwong@ucf.edu (L.T.); mona.shattell@ucf.edu (M.S.); 2 Research Development and Innovation Center, Our Lady of Fatima University, 120 McArthur Highway, Marulas, Valenzuela 1440, Philippines; crvillafuerte@fatima.edu.ph (C.M.V.); pbalbin@fatima.edu.ph (P.T.B.); 3 The Libraries, De La Salle University, 2401 Taft Avenue, Malate, Manila 0922, Philippines; luis.cruz@dlsu.edu.ph (L.E.D.C.); gerald.dino@dlsu.edu.ph (G.C.D.); 4 School of Nursing, Southern Institute of Technology, 133 Tay Street, Invercargill 9810, New Zealand; aica.rivero@sit.ac.nz; 5 Natural Science Department, Our Lady of Fatima University, 4176-A, MacArthur Highway, San Fernando 2000, Pampanga, Philippines; eamallo@fatima.edu.ph; 6 Nicholson School of Communication and Media, University of Central Florida, 2405 Aquarius Agora Dr, Orlando, FL 32816, USA; animator@ucf.edu

**Keywords:** virtual interactive agents, avatar, remote care, telehealth, older adults, systematic review, network analysis

## Abstract

**Background:** The global rise in the aging population presents significant challenges to healthcare systems, especially with increasing rates of chronic illnesses, mental health issues, and functional decline among older adults. In response, holistic and tech-driven approaches, such as telehealth and remote virtual interactive agents (VIAs), are potential emerging solutions to support the physical, cognitive, and emotional well-being of older adults. VIAs are multimodal digital tools that provide interactive and immersive experiences to users. Despite its promise, gaps still exist in the insights that explore ways of delivering geriatric healthcare remotely. **Objective:** This systematic review examines the existing literature on remote virtual interventions for older adults, focusing on bibliometrics, study purposes, outcomes, and network analysis of studies extracted from major databases using selected keywords and managed using the Covidence application. **Methods and Results:** Following five stages, namely, problem identification, a literature search, data evaluation, data analysis, and presentation, the review found that the studies on remote VIAs for older adults (2013–2025) were mostly from a positivist perspective, multi-authored, and U.S.-led, mainly showing positive outcomes for most studies (*n* = 13/15) conducted in home settings with healthy older participants. The dominance of positivist, US-led studies reflect an epistemological stance that emphasizes objectivity, quantification, and generalizability. VIAs, often pre-programmed and internet-based, supported health promotion and utilized visual humanoid avatars on personal devices. Keyword and network analysis additionally revealed four themes resulting from the review: Health and Clinical, Holistic and Cognitive, Home and Caring, and Hybrid and Connection. **Conclusions:** The review provides innovative insights and illustrations that may serve as a foundation for future research on VIAs and remote healthcare delivery for older adults.

## 1. Introduction

The aging process is a universal phenomenon [1]. Across the globe, the population of individuals aged 60 years or older is expected to more than double, increasing from 1 billion in 2020 to 2.1 billion in 2050 [2]. This trend is evident in most regions worldwide, wherein the older adult population, defined as those aged 60 and above [3], is projected to further outnumber the population of children younger than five years [4]. In a broader context, this demographic shift underscores the urgency of preparing and developing healthcare systems to meet the needs of a rapidly aging global population [5]. For various government and healthcare sectors, maintaining the overall health of older adults presents numerous challenges, particularly as age-related conditions like chronic illnesses and mental health issues continue to rise. Due to these alarming rates of disability that occur alongside increasing age, older adults often resort to substantial use of formal health services, consuming significant resources and rendering these systems inefficient and unsustainable in meeting their health needs [5].

As a response to these growing demands and limitations, health maintenance in aging populations requires a holistic and systematic approach that aims to go beyond treating diseases and symptoms and recognizes the interconnectedness among physical, cognitive, nutritional, and social aspects of geriatric health [6]. Older adults face a wide range of complex health challenges, other than physical issues, which mainly center on increased susceptibility to mental health disorders and social isolation. In line with this, around 14% of adults aged 60 and over live with a mental disorder, accounting for 10.6% of the total disability among older individuals. Additionally, nearly 95% of this population has at least one chronic condition, while approximately 80% have two or more [7]. This high prevalence of psychological and cognitive issues among older adults often results in mild short-term memory loss, word-finding difficulty, and slower processing speed, which all detrimentally affect the capacity to perform basic self-care and activities of daily living (ADLs). In the aspect of physical health, aging brings significant consequences, particularly with the reduction in functional independence and an increase in disability rates. These include sensory and urologic changes, muscle strength and fat changes, and somatic diseases like hypertension, osteoarthritis, and diabetes mellitus, among others [1]. Collectively, the occurrence of these conditions and limitations demonstrates an increasing concern in older adults’ independence and quality of life [8].

In addressing the need of older adults to maintain health in physical, social, and emotional terms, it is essential to emphasize improved strategies and interventions that explore the applications of modern technology to improve the quality of life. It has been observed over the years that the definition of well-being has evolved from traditional biological, mental, and psychological states to virtual and technological states that involve healthy interactions in non-physical techno-social realities [9]. The recent advent of digitalization has especially facilitated novel avenues for healthcare delivery and support, specifically through tools such as health-monitoring devices, assistive technologies, and digital platforms that aim to provide a broader range and more patient-centric approaches to transforming modern healthcare and bettering health management procedures [10]. Telehealth is a specific form of this technology, which supports electronic methods and information that can be used to remotely connect specialists to patients and providers for assessment and supplementation of in-person services [11]. With the emergence of these digital entities, healthcare systems are now gradually shifting focus toward proactive and personalized care that has the capacity to improve the well-being and autonomy of older adults [12].

Telehealth platforms integrate virtual interactive agents (VIAs), one of the various modern technologies that have been gaining increasing attention due to their capacity to provide profound assurance and versatile functions for more efficient methods of setting up public services and healthcare. VIAs are multimodal digital tools that offer authentic interaction experiences with the users [13], commonly with avatar representations or a textual interface, such as embodied conversational agents [14]. Building on different innovative ways to provide healthcare at a distance, remote VIAs show promising potential to enhance the health and lifestyle of older adults; however, a more comprehensive understanding and emerging insights are needed to further advance the existing knowledge in this field. To address this gap, this systematic review aims to critically examine published studies on remote VIAs for older adults. It seeks to uncover the characteristics of papers published, assess the progression and current state of this domain, develop a model, and provide insights to inform theory, practice, and future research directions. Specifically, the objectives of this review are (1) to describe the bibliometrics of articles published related to VIAs for older adults, (2) to analyze the purpose and outcomes of the included literature, (3) to map the attributes and current state of the art and science of VIAs in geriatric care, and (4) to generate topic clusters based on article keywords related to virtual agents for older adults. This study employed a network analysis and literature review, with the former conducted before the review, in order to quantitatively map relationships among authorship, keywords, and themes for identifying emerging patterns in the field. The latter subsequently synthesized the content of the studies to provide more in-depth and qualitative insights into thematic developments.

## 2. Materials and Methods

A systematic review was employed to evaluate the attributes of telehealth-enabled VIAs in the broad range of approaches for modern geriatric healthcare. This followed the stages outlined and developed by Whittemore and Knafl (2005) [15], focusing on five stages: (a) problem identification, (b) literature search, (c) data evaluation, (d) data analysis, and (e) presentation. Adherence to Preferred Reporting Items for Systematic Reviews and Meta-Analysis (PRISMA) will also be observed, alongside the refining of the literature search, article reviews, and collection of relevant data.

### 2.1. Stage 1: Problem Identification

Problem identification helps define specific areas that require further investigation, thereby guiding the development of relevant research questions. It is hypothesized that the utility and impact of VIAs for geriatric care are starting to emerge and address problems in healthcare; however, the science surrounding their applications to geriatric healthcare remains in its early stages, with various aspects of their definition, objectives, mechanisms, and outcomes remaining unclear. A comprehensive review of the existing literature is therefore crucial in determining patterns, features, benefits, and limitations in the field. Research questions were developed by considering four key elements: remote virtual interactive agents (concept), older adults aged 60 years and above (target population), geriatric care settings (context), and the current applications and frameworks of virtual agent technology (outcomes). This approach helped clarify the focus of the review and support a more targeted and effective search strategy. Aligned with these objectives, the following inquiries guided this systematic review: (1) What does the existing published literature describe about the use of VIAs? (2) What trends and patterns exist in terms of article purpose, outcomes, and description of VIAs? (3) What themes can be generated to describe the articles on VIAs?

### 2.2. Stage 2: Literature Search

On 16 June 2025, a comprehensive search strategy across five main databases and interfaces was implemented, namely Scopus, Web of Science, PubMed, ProQuest, and IEEE Xplore, which were selected for their coverage of interdisciplinary health sciences and technology that are most related to the use of VIAs in geriatric care. An initial exploratory search was conducted across various databases to identify similar studies, followed by a more extensive search using the identified databases and query strings, as presented in Table 1. To supplement database searches, the other literature was identified through handsearching techniques, such as a manual review of reference lists among all the included articles (i.e., backward citation searching). Moreover, a publication date limit was not imposed to ensure a thorough analysis of all the relevant literature; however, only English-language studies within the context of geriatric care were considered, with no geographic limitations. The search strategy is justified by the fact that the selected databases are recognized as leading sources in their respective fields, while the constructed search strings are compliant with their specific standards.

### 2.3. Stage 3: Data Evaluation

To evaluate the quality of the studies included in the review, a multi-step screening process was conducted using the Covidence (Melbourne, Australia) application, where the project was created on June 20, 2025. All the retrieved articles were uploaded, and duplicates were automatically removed. A minimum of two reviewers independently performed title and abstract screening, followed by a full-text review for the identification of eligibility based on defined inclusion and exclusion criteria. Any conflicts were resolved through group discussions facilitated by the principal investigator. Screening also considered areas such as title, abstract, authorship, and full-text content to reach a consensus. The inclusion criteria require that the articles discuss (1) virtual agents using (2) textual, audio, or visual (i.e., humanoid or non-humanoid) avatars and chatbots, (3) with health-related outcomes, whether physical, cognitive, emotional, or social, and (4) involving older adults aged 60 years and above.

**Figure 1:**
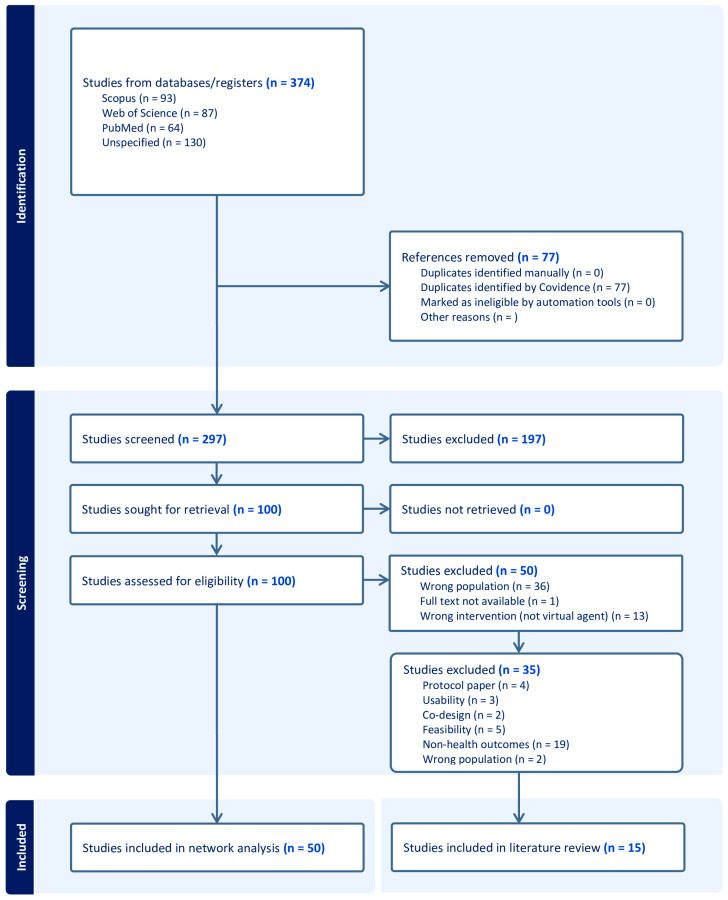
PRISMA diagram.

Additionally, studies that included adolescents and robotics, as well as review articles, were excluded. The Mixed Methods Appraisal Tool (MMAT, McGill, downloaded from https://www.mcgill.ca/familymed/research/resources/funding/mmat accessed on 4 on July 2025) was utilized to assess the methodological quality of the included studies and to minimize bias, ensuring the reliability of the results. Lastly, the study selection process was documented using a PRISMA flow diagram (Figure 1), which illustrates the number of records identified, screened, excluded, and included in the review, along with reasons for exclusions at each stage. The study protocol was registered at OSF Registries (https://osf.io/6ueht accessed on 15 June 2025).

Figure 1 illustrates the study identification, screening, eligibility assessment, and inclusion process conducted via Covidence. A total of 374 studies were included, with 77 duplicates removed. Among the 297 records screened, 197 were excluded at the title or abstract screening stage, as they did not fulfill the inclusion criteria discussed in stage 3. A full-text review was conducted for 100 articles, of which 50 were excluded due to incorrect population or intervention, unavailability of full-text, or lack of health outcomes, leaving 35 protocol papers. Ultimately, 50 studies were included in the network analysis and 15 in the literature review.

### 2.4. Stage 4: Data Analysis

A systematic and iterative process was followed in data analysis to identify patterns, relationships, and emerging themes across the included studies. A minimum of two authors independently extracted primary data, which was compared for consistency. In case of discrepancies, a group consensus was reached to ensure the accuracy and reliability of the data. The Covidence extraction interface was primarily utilized, through which data were transferred into a spreadsheet file after consensus. Afterwards, the data were organized using VOSviewer (version 1.6.20) to visualize co-authorship networks, keyword co-occurrence, and thematic clusters. Entities to be extracted specifically include authors, authorship, article type, journal type, country and region of origin, title, purpose, outcomes, keywords, year of publication, setting, subjects, sampling, condition/health status, project name, health dimension, representation, device, smart capability, connectivity, and features. Interrater reliability (Cohen’s κ = 0.66, PA = 84%) was computed using Covidence, which managed conflict resolution.

### 2.5. Stage 5: Presentation

**Figure 2:**
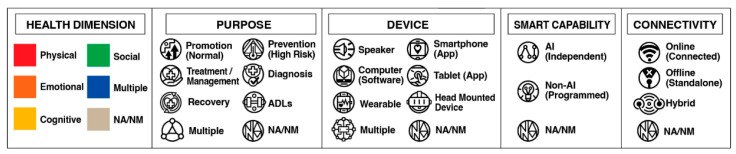
Labels and icons for technology features of VIAs.

In terms of synthesis and quality assessment, data were synthesized via the standards published [15] and publication bias was assessed using the MMAT and AACODS checklists. Spot-checking techniques were employed for data validation. The articles were coded and labeled based on selected technology features (Figure 2) found in the literature.

## 3. Results

### 3.1. Bibliometrics

**Figure 3:**
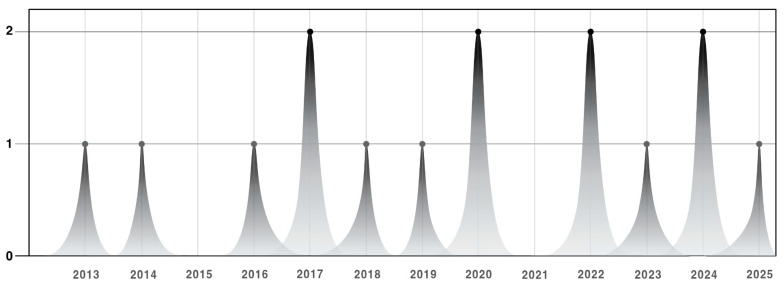
Number of VIA publications from 2013–2025.

A total of 15 articles centering on the influence of telehealth-enabled VIAs in enhancing and supporting geriatric health and well-being were included in the bibliometric analysis. As shown (Table 2), most of these studies (93.33%) were found to be products of collaborative efforts involving multiple authors, indicating the cooperative nature of healthcare technology research. Only one of these articles (6.67%) had two authors, while a single author produced none. The included studies were also categorized based on article type (i.e., qualitative, quantitative, mixed methods research), with nearly half of these (46.67%) employing a quantitative design. Most publications came from health technology journals (53.33%) in the regions of America (40%) and Europe (33.33%). Patterns in the publication related to the use of VIAs in geriatric care exhibit a steady increase from 2013 to 2025, with the years 2017, 2020, 2022, and 2024 having the highest occurrences (Figure 3).

### 3.2. Article Purpose and Outcomes

The findings from 15 articles exploring the utilization of telehealth-enabled VIAs in older adults were synthesized through a systematic review. Two core domains were extracted from the articles: (1) the purpose of the intervention and (2) the reported outcomes. The purpose and outcomes are presented in Table 3, while the patterns that emerged are thematically presented in Table 4. In terms of the purpose, interventions were classified into five categories: (1) impact on emotional and social well-being, (2) impact on health promotion and behavior change (26%), (3) impact on activities of daily living, (4) identify user perception and engagement, and (5) investigate VIA usability. On the other hand, the outcome of the interventions is (1) significant reductions in loneliness, mood improvement, and emotional satisfaction, (2) improved exercise routines, better adherence to health advice, and motivation, (3) helped maintain emotional and cognitive engagement, (4) positive perception and increased engagement with VIAs, and (5) enhanced usability and acceptance. More than half of the articles (*n* = 8; 53.33%) were of a hundred percent MMAT quality, while the remaining were at 80% (*n* = 7; 46.66%).

### 3.3. Health Dimensions and Technology Characteristics of Virtual Interactive Agents for Older Adults

**Figure 4:**
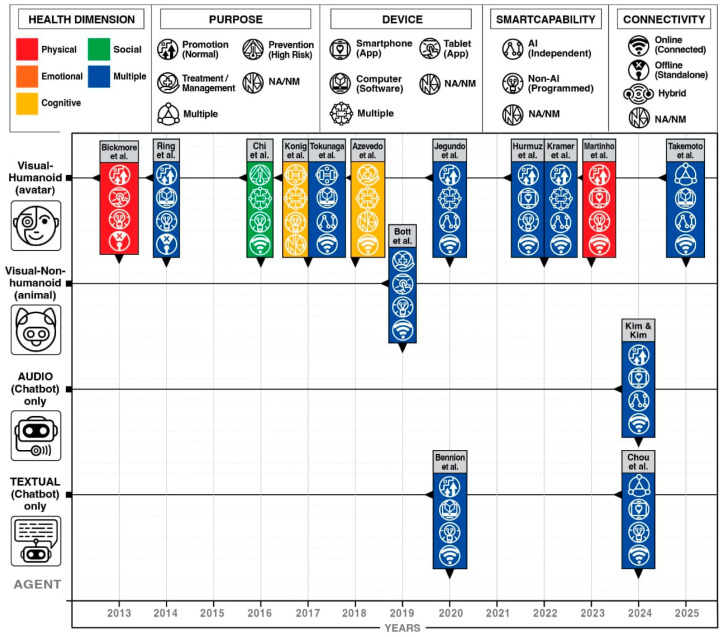
Evolution, health dimensions, and technology characteristics of virtual interactive agents for older adults, Bickmore et al. [17], Ring et al. [24], Chi et al. [22], Konig et al. [23], Tokunaga et al. [30], Azevedo et al. [28], Bott et al. [16], Jegundo et al. [21], Bennion et al. [25], Hurmuz eat al [19], Kramer at al [26], Martinho et al. [18], Kim & Kim [20], Chou et al. [27], Takemoto et al. [29].

The health dimensions and technology characteristics of VIAs are visualized in Figure 4 (see Supplementary Materials Table S1). A significant portion of the articles have utilized telehealth-enabled VIAs for health promotion (*n* = 8; 53.33%), while a small portion were used for treatment (*n* = 2; 13.33%), ADLs (*n* = 2; 13.33%), and disease prevention (*n* = 1; 6.66%). There are also two studies that were used for multiple purposes (*n* = 2; 13.33%). Regarding various health domains where VIAs were used, most articles employed them for multiple purposes (*n* = 10; 66.66%), while they were used sparingly for physical (*n* = 2; 13.33%), cognitive (*n* = 2; 13.33%), and social aspects (*n* = 1; 6.66%).

The remote VIAs were also described based on their agent characteristics, device used, smart capability, connectivity, and appearance. Most articles have utilized a visual humanoid avatar to communicate the VIAs to older adult users (*n* = 11; 73.33%). However, a handful used a textual chatbot (*n* = 2; 13.33%), an audio chatbot (*n* = 1; 6.66%), as well as a visual non-humanoid VIAs (*n* = 1; 6.66%). VIAs were mostly used through the aid of multiple devices (*n* = 5; 33.33%); however, some were based on smartphone (*n* = 4; 26.66%) and tablet applications (*n* = 2; 13.33%), as well as computer software (*n* = 4; 26.66%). The use of AI was also prominent in the obtained articles (*n* = 5; 33.33%); however, non-AI or programmed VIAs dominated the review (*n* = 9; 60%). One (6.66%) article did not mention any smart capabilities of their VIAs. In line with this, 11 articles (73.33%) used internet connectivity to function; however, three could be operated offline (20%). One article also did not mention any details about connectivity (6.66%). Furthermore, some articles did not provide documentation of their VIAs (*n* = 3; 20%), but the majority did (*n* = 11; 80%).

To map the evolution and characteristics of remote VIA use in older adult healthcare, Figure 4 illustrates a synthesis containing 15 studies from 2013 to 2025. This mainly focused on both the state of the art and science of VIAs for older adults, by which the former encompasses the form, interface, and presentation of agents, whereas the latter addresses their purposes, functionalities, and health impacts. A figure utilizing a Cartesian plane was constructed to create a visual representation of these trends, with the x-axis denoting the publication year and the y-axis categorizing agent modality (e.g., visual humanoid, visual non-humanoid, audio-only, textual-only). Each plotted point demonstrates a study and its accompanying icons or colors summarize six key attributes: (1) author/s, (2) health dimension addressed, (3) primary purpose of the article (e.g., promotion, prevention, treatment), (4) device/s used, (5) smart capability (i.e., AI-driven or non-AI-dependent), and (6) connectivity status (i.e., online or offline use).

In terms of the state of the art, visual humanoid agents or avatars with human-like appearances dominate the landscape, where such a domain was utilized in 11 out of the 15 studies. Conversely, non-humanoid avatars and audio- or text-only agents are less common in the studies. Other than visuals, the use of mobile apps on smartphones and tablets is prevalent among older adult populations, with more recent studies (e.g., 2020–2025) showing an increased shift toward AI-powered VIAs.

Patterns in the state of the science demonstrate that health promotion is the most frequently cited purpose across the studies, followed by treatment, or management, and prevention. The VIAs analyzed addressed multiple dimensions of health, including physical, cognitive, social, and emotional well-being. Earlier studies predominantly featured non-AI and standalone agents, while more recent articles highlighted the integration of AI capabilities and online connectivity. Lastly, connectivity emerged as a critical component, with most agents requiring internet access for full functionality.

### 3.4. Network Analysis–Keyword Co-Occurrence and Topic Clusters

**Figure 5:**
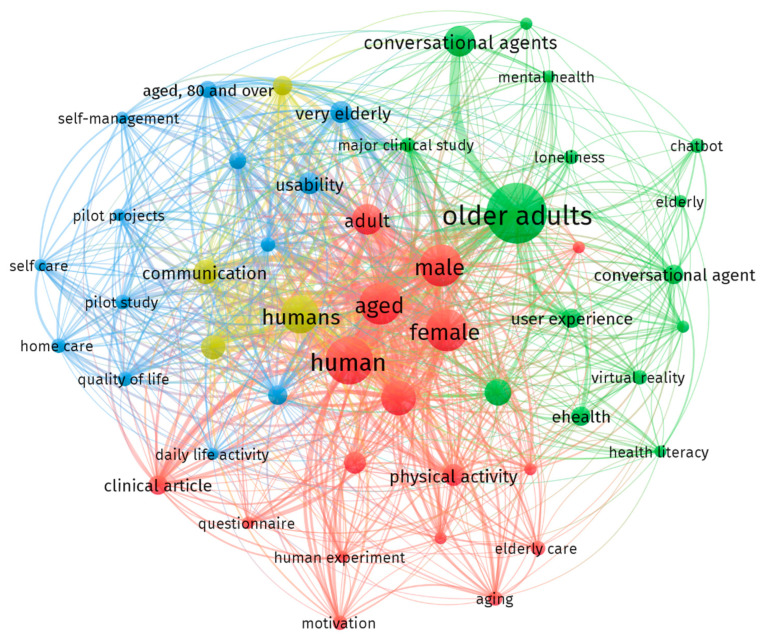
Article keywords: co-occurrence and topic clusters network map.

Figure 5 demonstrates the co-occurrence network of article keywords, revealing four key thematic clusters from the systematic review (Table 5) that illustrate the characteristics and attributes of research on telehealth-enabled VIAs for geriatric healthcare. The network revealed that “older adults”, “human”, “male” and “female”, and “conversational agents” are some of the most frequently occurring keywords in the map, thus conveying how key concepts are interconnected through node sizes that represent word frequency and line thickness for co-occurrence strength. Four keyword clusters, namely, (1) Health and Clinical, (2) Holistic and Cognitive, (3) Home and Caring, and (4) Hybrid and Connection, were additionally identified. The first group encompasses studies on how VIAs can motivate and support health through physical and behavioral aspects, while the second addresses other dimensions of health such as literacy, cognitive, and mental health support. The third cluster, Home and Caring, includes evaluative studies emphasizing the effectiveness of VIAs in supporting independent living, aging-in-place, self-care management, and quality of life. The last cluster explored how the agents facilitate interpersonal communication patterns across different adult age cohorts. The observed pattern illustrates how the existing literature on remote VIAs for geriatric care is multi-dimensional, spanning physical and mental health, alongside technological innovation and social support.

## 4. Discussion

### 4.1. Bibliometrics

Fifteen articles were included in the review’s bibliometric analysis. Most of these studies were published in health technology journals, often under multiple authorship, and primarily employed a positivist approach. This trend reflects a broader shift in digital health research toward interdisciplinary approaches that emphasize generalizability and quantification to support wider application across larger populations, and align with current positivist paradigms in health technology research [31]. In addition, the prevalence of multi-authorship papers among various health technology journals underscores the cross-sector and collaborative nature of developing remote VIAs for geriatric care. These observations raise the need for a qualitative, user-centered approach to research in order to capture the nuanced experiences, preferences, and ethical considerations of the older adult population [32].

Results also showed that few articles were published between 2013 and 2025. This may be attributed to VIA design and testing being highly specialized, new and emerging, and resource-intensive fields that require substantial time, funding, and interdisciplinary work [33,34]. This finding suggests that the observable limitation in the number of publications related to VIAs and older adults across these years reflects a lag in research capacity and integration within the broader fields of gerotechnology. A pressing need is highlighted for targeted funding, interdisciplinary programs, and partnerships to advance this area.

Lastly, the United States generated the greatest number of articles related to the use of remote VIAs in older adult healthcare. This may stem from the fact that the United States possesses a strong research infrastructure, higher funding availability, and a more advanced technology industry [35]. In addition, the population of countries in the region, such as the United States, is increasingly aging [36], which creates higher demand for other health service delivery modalities such as VIAs. This highlights how factors such as demographic pressures and institutional capacities are capable of driving innovation in aging-related technologies [37], but also implies a geographic concentration in research, possibly suggesting a potential gap in the studies of telehealth-enabled VIA among low- and middle-income countries where populations are also aging but research infrastructure may be limited [38].

### 4.2. Article Purpose and Outcomes

The outcomes of the articles communicate the positive impact of VIAs for older adults, with few studies having neutral results. Potential associations may be found in how VIAs in health service delivery for older adults are a promising field [39] and, on the other hand, how healthy and technologically inclined older adults could provide more favorable responses, corresponding to a phenomenon called respondent bias [40]. These imply that while current evidence supports the capacity of VIAs in enhancing geriatric health service delivery, the existing predominance of positive outcomes must be interpreted with awareness of potential limitations. The presence of respondent bias, for instance, may lead to overestimation of VIA effectiveness and acceptability, thus underscoring the need for more diverse and representative sampling methods in future studies.

In the reviewed studies, research articles involved an average of 50 healthy older adult participants selected via non-probability sampling techniques. Participants were typically recruited from senior centers, communities, and clinics, rather than through random sampling methods. This recruitment approach may introduce bias [41], as older adults who are more accessible through these areas may tend to be healthier and more socially active. Other older adult participants may also not be technologically inclined, which can lower both response and retention rates in technology-based interventions. To reduce variability and simplify implementation, many studies preferred healthier or cognitively normal older adult cohorts. This decision reflects not only practical challenges but also the increased ethical considerations and procedural requirements involved in sampling older adults with medical or cognitive conditions [42,43]. To ensure that results on remote VIA interventions are equitable and more generalizable, future studies must prioritize more representative sampling strategies and ethical frameworks that facilitate safe inclusion of medically or cognitively vulnerable populations.

The review revealed that experiments on VIAs for older adults were primarily conducted in community or home settings, with a few also taking place in medical and research facilities. This implies that current research in the field has primarily been situated in naturalistic environments where older adults live and interact, providing accessibility and comfort to the participants [44,45]. Future studies may consider examining whether such settings influence the outcomes of VIA interventions, especially in relation to accessibility and participation of individuals with mobility or health limitations.

### 4.3. Health Dimensions and Technology Characteristics of Virtual Interactive Agents for Older Adults

Virtual agents are mostly utilized in health promotion activities covering multiple health domains (i.e., physical, cognitive, emotional, and social), reflecting both the complex health challenges faced by older adults and the versatility of VIA technology. Because multiple health issues, such as chronic diseases, mental health issues, and age-related changes, challenge older adults every day [4], this may indicate that the scalability and adaptability of telehealth-enabled VIAs make these particularly useful in complementing traditional healthcare services that aim to tailor problems in different areas of health, also aiding in the alleviation of burden on caregivers and health systems.

A considerable amount of VIAs were presented to older adults via visual humanoid avatars. This occurrence may be attributed to the Uncanny Valley Theory [46], which is a concept in robotics and human–computer interaction that discusses how the human-likeness of robots can increase users’ comfort and emotional affinity to these media up to a certain degree. Future studies are needed to determine how humanoid avatars can influence not only emotional responses, but also long-term adherence to healthcare interventions among older adults.

Considering the technical aspects, mobile devices and personal computers were the most common technology platforms for remote VIAs. Given the diverse cognitive and sensory needs of older adults, multimodal communication is necessary to ensure better engagement and comprehension. Notably, older adults tend to favor smartphones for both social and non-social purposes. For social use, news reading and social media are among the most common activities, while non-social motivations often include the prevention of loneliness and cognitive decline [47]. This also relates to a study finding that older adults identify their technology preferences based on personal experiences and a combination of various attributes [48].

In addition, results showed that most VIAs are pre-programmed and lack AI integration, although internet connectivity powered the majority of these technologies. Prior research has shown that the development of non-AI, rule-based systems is generally cheaper and easier to implement, as it does not require as enormous programming or complex machine learning models compared to its counterpart [49]. While AI integration remains an emerging and dynamic field, it introduces new complexities that are not yet widely adopted. The prevalence of pre-programmed VIAs reflects their low cost and reliability; however, the rising demand for personalized care may accelerate AI adoption [50].

### 4.4. Network Analysis–Keyword Co-Occurrence and Topic Clusters

Results of the study in this area highlight the multidimensional nature of the current literature focusing on VIAs for older adult healthcare, revealing four major thematic clusters: Health and Clinical, Holistic and Cognitive, Home and Caring, and Hybrid and Connection. The first cluster, concerning health and clinical applications, conveys that physical and behavioral health (e.g., social isolation) are addressed and promoted via the use of VIAs. The prominence of health and clinical applications suggests that VIAs can serve as valuable tools for promoting key health outcomes, while also highlighting their role in geriatric care by fulfilling social and educational functions aimed at supporting overall health and well-being [51]. Moreover, the holistic and cognitive cluster expands VIA functions to support literacy, mental health, cognitive functioning, and loneliness reduction. Virtual agents are shown to improve cognitive function in older adults by promoting social interaction and physical activity. Designing agents that support these purposes may enhance mental resilience and delay cognitive decline, especially for isolated or less tech-savvy users [52].

Other than the two identified clusters, findings in the Home and Caring category exhibit the primary role of VIAs in enabling aging-in-place, self-care, and independent living. Their ability to provide consistent, in-home support may reduce reliance on institutional care and allow for more proactive roles of the elderly in their overall health management and functional independence [53]. Lastly, the cluster of Hybrid and Connection highlights the emerging social function of VIAs in promoting interpersonal communication and reducing loneliness, especially across older adult groups. As social isolation remains a critical issue in older populations, VIAs have the potential to act as social companions or facilitators of connection [54]. Overall, the thematic findings generated from the reviewed literature indicate that VIAs are evolving into multifunctional tools that extend beyond simple task assistance and support the holistic health requirements among older adults.

## 5. Study Limitations

The results of this review must be interpreted based on several limitations. The outcomes of the study were reported from a pool of peer-reviewed articles written in the English language and intentionally excluded publications from social media and non-indexed work from other emerging information dissemination platforms (e.g., blog posts, social media for science), which future researchers may find relevant. Also, the network of keyword co-occurrences was developed based on indexed and author keywords using software with no AI integration. Future studies may wish to process bibliometrics data with other software and data analytics tools with advanced and smart features. Themes were generated cognizant of the healthcare field, and other researchers may want to extend their interpretation to other fields of inquiry (e.g., computer science, human–computer interaction).

## 6. Conclusions

The present work presents a synthesized perspective on the evolution, applications, and the state of science in the use of virtual interactive agents for geriatric care by yielding four core findings. First, the review involved studies on remote VIAs for geriatric care, which were published between 2013 and 2025 across health technology journals, followed a positivist approach, involved multiple authorship, and were predominantly U.S.-based. Secondly, studies were primarily conducted in community or home settings with an average of 50 healthy older adult participants selected through non-probability sampling, and they reported predominantly positive outcomes on the impact of VIAs, with a few yielding neutral results. Third, remote VIAs were observed to be primarily used for health promotion across physical, cognitive, emotional, and social domains, commonly delivered through visual humanoid avatars on mobile devices or personal computers, with most being pre-programmed, lacking AI integration, and powered by internet connectivity. Lastly, network analysis for keyword co-occurrence revealed four key thematic clusters, namely: Health and Clinical, Holistic and Cognitive, Home and Caring, and Hybrid and Connection.

Future projects detailing the ethical deployment of VIAs with consideration of client data privacy and consent may provide essential information on ethics-related policies for VIA integration in healthcare. A more targeted approach focusing on low-resource settings, longitudinal studies, or integration with other smart health systems will be crucial. Future research may also explore the impact of next-generation immersive technologies. For instance, studies could incorporate hyper-realistic virtual humans (e.g., Epic’s MetaHuman), to rigorously test existing theories (e.g., Uncanny Valley). These studies could be combined with fully immersive, gamified platforms, such as VR fitness applications, to move beyond the 2D screen-based interventions. This review offers foundational insights for future research on the growing health needs of the aging population and its potential reliance on health technology to address aging concerns.

## Figures and Tables

**Table 1:** Database search strategy.

Database	Search String
Scopus	TITLE-ABS-KEY ((“virtual companion” OR “virtual agent” OR “virtual avatar” OR “conversational agent”) AND (“older adults” OR elderly OR seniors OR “aging population”)) AND (LIMIT-TO (DOCTYPE, “ar”)) AND (LIMIT-TO (LANGUAGE, “English”)) AND (LIMIT-TO (SUBJAREA, “MEDI”) OR LIMIT-TO (SUBJAREA, “SOCI”) OR LIMIT-TO (SUBJAREA, “PSYC”) OR LIMIT-TO (SUBJAREA, “NURS”) OR LIMIT-TO (SUBJAREA, “HEAL”) OR LIMIT-TO (SUBJAREA, “BIOC”))
Web of Science	TS = ((“virtual companion” OR “virtual agent” OR “virtual avatar” OR “conversational agent”) AND (“older adults” OR elderly OR seniors OR “aging population”))
PubMed	((“virtual” [All Fields] OR “virtuality” [All Fields] OR “virtualization” [All Fields] OR “virtualized” [All Fields] OR “virtualizing” [All Fields] OR “virtuals” [All Fields]) AND (“companion *” [All Fields] OR “agent *” [All Fields] OR “avatar *” [All Fields]) AND “older adults” [All Fields]) AND (English [Filter]))
ProQuest	TX(“virtual”) AND TX(“companion *” OR “avatar” OR “agent”) AND SU(“older adults” OR elderly OR seniors) AND LA(English) AND STYPE(“scholarly journals”)
IEEE Xplore	(virtual) AND (companion * OR agent OR avatar) AND (“older adults” OR elderly OR senior * OR geriatric * OR “aging population”)

**Table 2:** Article bibliometrics.

Attributes	f	%
Authorship		
Single	0	0.00
Double	1	6.67
Multiple	14	93.33
Type		
Qualitative	3	20.00
Quantitative	7	46.67
Mixed	5	33.33
Journal Publication		
Health	4	26.67
Technology	3	20.00
Health Technology	8	53.33
Region		
America	6	40.00
European	5	33.33
Southeast Asia	0	0.00
Western Pacific	4	26.67
African	0	0.00
East Mediterranean	0	0.00

**Table 3:** Article purpose and outcomes.

No.	Authors	Title	MMAT *	Purpose	Outcomes
1	Bott et al. (2019) [16]	A Protocol-Driven, Bedside Digital Conversational Agent to Support Nurse Teams and Mitigate Risks of Hospitalization in Older Adults: Case Control Pre-Post Study	80%	To determine the influence of a bedside ECA on the psychological health and fall risk of hospitalized older adults.	The findings demonstrated that the bedside ECA is valid for use of hospitalized older adults.
2	Bickmore et al. (2013) [17]	A Randomized Controlled Trial of an Automated Exercise Coach for Older Adults	80%	To compare an ECA connected to a pedometer against the traditional use of a pedometer in sedentary older adults.	Walking among the participants has increased.
3	Martinho et al. (2023) [18]	Effects of a Gamified Agent-Based System for Personalized Elderly Care: Pilot Usability Study	80%	To assess the effectiveness of the gamified agent-based system in older adults’ physical activity.	After seven days, the step count of the participants increased.
4	Hurmuz et al. (2022) [19]	Evaluation of a virtual coaching system eHealth intervention: A mixed methods observational cohort study in the Netherlands	100%	To assess the potential health effects of the Council of Coaches, a CA-based eHealth platform, in the real world	Using COUCH can aid in motivating older adults to adopt a healthier lifestyle.
5	Kim & Kim (2024) [20]	Experience of the Use of AI Conversational Agents Among Low-Income Older Adults Living Alone	100%	To explore the benefits of Arya to older adults	Two perceived benefits were recorded: instrumental/functional and emotional
6	Jegundo et al. (2020) [21]	Perceived Usefulness, Satisfaction, Ease of Use and Potential of a Virtual Companion to Support the Care Provision for Older Adults	100%	To assess older adults’ perceptions of an agent’s usefulness, satisfaction, and ease of use, and to explore its potential for care provision from the perspective of formal caregivers.	The results of both the observational study and the focus group revealed good perceptions on the role of virtual companions to support care for older adults.
7	Chi et al. (2017) [22]	Pilot testing a digital pet avatar for older adults	100%	To examine perceived acceptance and utility of a tablet-based human-controlled system with a pet avatar used by older adults	Older adults found the digital pet avatar generally enjoyable and beneficial for companionship, despite concerns about limited conversational ability, technical issues, and privacy.
8	Konig et al. (2017) [23]	Qualitative study of affective identities in dementia patients for the design of cognitive assistive technologies	100%	To develop an emotionally intelligent cognitive assistant (ICA) to help older adults with Alzheimer’s in completing their ADLs.	Each participant holds multiple identities (e.g., father, husband). While memory-based identities may fade, habitual aspects of one’s persona often persist even without context.
9	Ring et al. (2015) [24]	Social support agents for older adults: longitudinal affective computing in the home	100%	To evaluate the feasibility and effectiveness of an in-home conversational agent for reducing loneliness in older adults.	The proactive conversational agent significantly reduced loneliness and improved mood among older adults.
10	Bennion et al. (2020) [25]	Usability, Acceptability, and Effectiveness of Web-Based Conversational Agents to Facilitate Problem Solving in Older Adults: Controlled Study	100%	To compare and contrast the system usability of 2 chatbots (MYLO and ELIZA) in an older adult sample	MYLO users spent more time with the agent, reported less distress, found it more helpful, and showed high adherence.
11	Kramer et al. (2022) [26]	Use and Effect of Embodied Conversational Agents for Improving Eating Behavior and Decreasing Loneliness Among Community-Dwelling Older Adults: Randomized Controlled Trial	80%	To assess the effects of ECAs on diet, loneliness, and factors influencing their use among older adults.	The study found no clear links between ECA use and health effects but suggests future designs should align with users’ readiness to change.
12	Chou et al. (2024) [27]	User-Friendly Chatbot to Mitigate the Psychological Stress of Older Adults During the COVID-19 Pandemic: Development and Usability Study	80%	To examine whether a chatbot can mitigate psychological stress among older adults with anxiety or depressive disorders during the COVID-19 pandemic.	There is a a significant reduction in loneliness among participants aged ≥65 years after using the chatbot for 4 weeks.
13	Azevedo et al. (2018) [28]	Using conversational agents to explain medication instructions to older adults	80%	To assess how conversational agents can improve older adults’ understanding and recall of medication instructions.	Older adults showed better recall and more positive responses to gain-framed messages from realistic conversational agents.
14	Takemoto et al. (2025) [29]	Virtual avatar communication task eliciting pseudo-social isolation and detecting social isolation using non-verbal signal monitoring in older adults	100%	To develop a virtual avatar conversation cyberball task and evoke pseudosocial isolation in older adults, and to identify non-verbal indicators that reflect social isolation.	Pseudosocial isolation in older adults can be effectively induced through a virtual avatar that detects non-verbal indicators like blink frequency and eye muscle movement.
15	Tokunaga et al. (2017) [30]	VirtualCareGiver: Personalized Smart Elderly Care	100%	To develop and evaluate a personalized virtual caregiver system that provides individualized care for older adults through cloud-based smart services and virtual agents.	Personalized virtual care supported older adults’ emotional well-being and cognitive engagement.

* MMAT—Mixed Methods Appraisal Tool.

**Table 4:** Thematized purpose and outcomes of VIA publication.

ARTICLE GROUPS				
[16,17,18,19]	[20,21,22,23]	[24,25,26]	[27,28]	[27,28,29]
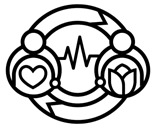	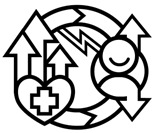	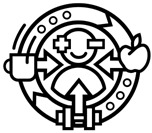	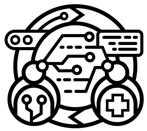	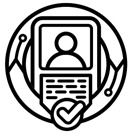
PURPOSE				
Impact on Emotional and Social Well-being	Impact on Health Promotion and Behavior Change	Impact on Activities of Daily Living	Identify User Perception and Engagement	Investigate VIA Usability
OUTCOMES				
Significant reductions in loneliness, mood improvement, and emotional satisfaction.	Improved exercise routines, better adherence to health advice, and motivation.	Helped maintain emotional and cognitive engagement.	Positive perception and increased engagement with VIAs.	Enhanced Usability and Acceptance.

Note: Numbers in brackets indicate the range of articles included in each thematic group. Icons are illustrative and represent the focus of the studies. Rows labeled “Purpose” summarize the aims of the publications, while rows labeled “Outcomes” summarize their reported findings.

**Table 5:** Virtual interactive agents article keywords: clusters with grouped keywords according to themes.

Cluster	Keywords	Theme	Description
1	adult, aged, aging, article, clinical article, computer interface, elderly care, exercise, female, human, human experiment, male, motivation, physical activity, questionnaire, social isolation, voice	Health and Clinical	Research examining how virtual agents can motivate and support health in terms of physical exercise and behavioral change interventions while addressing social isolation
2	artificial intelligence, chatbot, conversational agent, conversational agents, eHealth, elderly, embodied conversational agent, health literacy, health promotion, loneliness, major clinical study, mental health, older adults, user experience, virtual reality	Holistic and Cognitive	Studies investigating advanced technologies in virtual agents, including embodied agents and VR applications for promoting other dimensions of health (holistic) including literacy, cognitive and mental health support, and loneliness reduction.
3	Aged 80 and over, controlled study, daily life activity, home care, pilot projects, pilot study, procedures, quality of life, randomized controlled trial, self-care, self-management, usability, very elderly	Home and Caring	Rigorous experimental research evaluating virtual agent effectiveness in supporting independent living and aging-in-place support, self-care management, and quality of life improvement through controlled trials and usability studies.
4	Communication, humans, interpersonal communication, middle aged	Hybrid and Connection	Research exploring how virtual agents facilitate and enhance interpersonal communication patterns and social interaction across different adult age cohorts

Note: Keywords were manually coded and clustered by similarity; overarching themes and descriptions were derived from these groupings.

## Data Availability

No new data were created or analyzed in this study. Data sharing is not applicable to this article.

## Supplementary Materials

The following supporting information can be downloaded at https://www.mdpi.com/article/10.3390/healthcare13172253/s1; Table S1. Health dimensions and technology characteristics of virtual interactive agents for older adults.

## Abbreviations

The following abbreviations are used in this manuscript:

VIA	Virtual Interactive Agent
MMAT	Mixed Method Appraisal Tool

## References

[1] Jaul E., Barron J. (2017). Age-Related Diseases and Clinical and Public Health Implications for the 85 Years Old and Over Population. Front. Public Health.

[2] World Health Organization Ageing: Global Population. https://www.who.int/news-room/questions-and-answers/item/population-ageing.

[3] Rees P. (2009). Demography. International Encyclopedia of Human Geography.

[4] World Health Organization Ageing and Health. https://www.who.int/news-room/fact-sheets/detail/ageing-and-health.

[5] Jones C.H., Dolsten M. (2024). Healthcare on the Brink: Navigating the Challenges of an Aging Society in the United States. npj Aging.

[6] Kotsani M., Kravvariti E., Avgerinou C., Panagiotakis S., Bograkou Tzanetakou K., Antoniadou E., Karamanof G., Karampeazis A., Koutsouri A., Panagiotopoulou K. (2021). The Relevance and Added Value of Geriatric Medicine (GM): Introducing GM to Non-Geriatricians. J. Clin. Med..

[7] World Health Organization Mental Health of Older Adults. https://www.who.int/news-room/fact-sheets/detail/mental-health-of-older-adults.

[8] Noto S. (2023). Perspectives on Aging and Quality of Life. Healthcare.

[9] Dino M.J., Vital J.C., Patricio C., Catajan M.W., Ong I., Gallardo A., Macaspac R., De Vera O., Santos F., Agustin P.D. (2023). Charting the Uncharted: Mapping Scientific Publications on Online Disinhibition Effect in the Digital Space via Bibliometrics and Network Analyses. Comput. Hum. Behav. Rep..

[10] Stoumpos A.I., Kitsios F., Talias M.A. (2023). Digital Transformation in Healthcare: Technology Acceptance and Its Applications. Int. J. Environ. Res. Public Health.

[11] La Valle C., Johnston E., Tager-Flusberg H. (2022). A Systematic Review of the Use of Telehealth to Facilitate a Diagnosis for Children with Developmental Concerns. Res. Dev. Disabil..

[12] Bogoslov I.A., Corman S., Lungu A.E. (2024). Perspectives on Artificial Intelligence Adoption for European Union Elderly in the Context of Digital Skills Development. Sustainability.

[13] Shaked N.A. (2017). Avatars and Virtual Agents—Relationship Interfaces for the Elderly. Healthc. Technol. Lett..

[14] Kyrlitsias C., Michael-Grigoriou D. (2022). Social Interaction with Agents and Avatars in Immersive Virtual Environments: A Survey. Front. Virtual Real..

[15] Whittemore R., Knafl K. (2005). The Integrative Review: Updated Methodology. J. Adv. Nurs..

[16] Bott N., Wexler S., Drury L., Pollak C., Wang V., Scher K., Narducci S. (2019). A Protocol-Driven, Bedside Digital Conversational Agent to Support Nurse Teams and Mitigate Risks of Hospitalization in Older Adults: Case Control Pre-Post Study. J. Med. Internet Res..

[17] Bickmore T.W., Silliman R.A., Nelson K., Cheng D.M., Winter M., Henault L., Paasche-Orlow M.K. (2013). A Randomized Controlled Trial of an Automated Exercise Coach for Older Adults. J. Am. Geriatr. Soc..

[18] Martinho D., Vítor Crista Carneiro J., Matsui K., Corchado J.M., Marreiros G. (2023). Effects of a Gamified Agent-Based System for Personalized Elderly Care: Pilot Usability Study. JMIR Serious Games.

[19] Hurmuz M.Z.M., Jansen-Kosterink S.M., Beinema T., Fischer K., op den Akker H., Hermens H.J. (2022). Evaluation of a virtual coaching system eHealth intervention: A mixed methods observational cohort study in the Netherlands. Internet Interv..

[20] Kim K., Kim S. (2024). Experience of the Use of AI Conversational Agents Among Low-Income Older Adults Living Alone. SAGE Open.

[21] Jegundo A.L., Dantas C., Quintas J., Dutra J., Almeida A.L., Caravau H., Rosa A.F., Martins A.I., Pacheco Rocha N. (2020). Perceived Usefulness, Satisfaction, Ease of Use and Potential of a Virtual Companion to Support the Care Provision for Older Adults. Technologies.

[22] Chi N.-C., Sparks O., Lin S.-Y., Lazar A., Thompson H.J., Demiris G. (2017). Pilot testing a digital pet avatar for older adults. Geriatr. Nurs..

[23] König A., Francis L.E., Joshi J., Robillard J.M., Hoey J. (2017). Qualitative study of affective identities in dementia patients for the design of cognitive assistive technologies. J. Rehabil. Assist. Technol. Eng..

[24] Ring L., Shi L., Totzke K., Bickmore T. (2015). Social support agents for older adults: Longitudinal affective computing in the home. J. Multimodal User Interfaces.

[25] Bennion K.A., Tate D., Muñoz-Christian K., Phelan S., Muñoz-Christian K. (2020). Impact of an Internet-Based Lifestyle Intervention on Behavioral and Psychosocial Factors During Postpartum Weight Loss. Obesity.

[26] Kramer L., van Velsen L., Clark J., Mulder B., de Vet E. (2022). Use and Effect of Embodied Conversational Agents for Improving Eating Behavior and Decreasing Loneliness Among Community-Dwelling Older Adults: Randomized Controlled Trial. JMIR Form. Res..

[27] Chou Y.-H., Lin C., Lee S.-H., Lee Y.-F., Cheng L.-C. (2024). User-Friendly Chatbot to Mitigate the Psychological Stress of Older Adults During the COVID-19 Pandemic: Development and Usability Study. JMIR Form. Res..

[28] Azevedo R.F.L., Morrow D., Graumlich J., Willemsen-Dunlap A., Hasegawa-Johnson M., Huang T.S., Gu K., Bhat S., Sakakini T., Sadauskas V. (2018). Using conversational agents to explain medication instructions to older adults. AMIA Annu. Symp. Proc..

[29] Takemoto A., Iwamoto M., Yaegashi H., Yun S., Takashima R. (2025). Virtual avatar communication task eliciting pseudo-social isolation and detecting social isolation using non-verbal signal monitoring in older adults. Front. Psychol..

[30] Tokunaga S., Tamamizu K., Saiki S., Nakamura M., Yasuda K. (2017). VirtualCareGiver: Personalized Smart Elderly Care. Int. J. Softw. Innov..

[31] Regan E.A. (2022). Changing the Research Paradigm for Digital Transformation in Healthcare Delivery. Front. Digit. Health.

[32] Safari K., McKenna L., Davis J. (2023). Promoting Generalisation in Qualitative Nursing Research Using the Multiple Case Narrative Approach: A Methodological Overview. J. Res. Nurs..

[33] Bulgaro A., Liberman-Pincu E., Oron-Gilad T. (2022). Bridging the Gap: Generating a Design Space Model of Socially Assistive Robots (SARs) for Older Adults Using Participatory Design (PD). arXiv.

[34] Chen C., Johnson J.G., Charles K., Lee A., Lifset E.T., Hogarth M., Moore A.A., Farcas E., Weibel N. Understanding Barriers and Design Opportunities to Improve Healthcare and QOL for Older Adults through Voice Assistants. Proceedings of the 23rd International ACM SIGACCESS Conference on Computers and Accessibility.

[35] Onken J., Aragon R., Calcagno A.M. (2019). Geographically-Related Outcomes of U.S. Funding for Small Business Research and Development: Results of the Research Grant Programs of a Component of the National Institutes of Health. Eval. Program Plann..

[36] Mather M., Scommegna P. Fact Sheet: Aging in the United States. https://www.prb.org/resources/fact-sheet-aging-in-the-united-states/.

[37] Bergschöld J.M., Gunnes M., Eide A.H., Lassemo E. (2024). Characteristics and Range of Reviews About Technologies for Aging in Place: Scoping Review of Reviews. JMIR Aging.

[38] Saha I., Sundström C., Kandasamy A., Kraepelien M., Dahiya N., Saha A., Jayaram-Lindström N., Chakrabarti A., Benegal V. (2025). Digital Interventions for Common Mental Health Problems among Older Adults in Low- and Middle-Income Countries: A Scoping Review. BMJ Glob. Health.

[39] Amjad A., Kordel P., Fernandes G. (2023). A Review on Innovation in Healthcare Sector (Telehealth) through Artificial Intelligence. Sustainability.

[40] An J., Zhu X., Wan K., Xiang Z., Shi Z., An J., Huang W. (2024). Older Adults’ Self-Perception, Technology Anxiety, and Intention to Use Digital Public Services. BMC Public Health.

[41] Boyd R.J., Powney G.D., Pescott O.L. (2023). We Need to Talk about Nonprobability Samples. Trends Ecol. Evol..

[42] Forsat N.D., Palmowski A., Palmowski Y., Boers M., Buttgereit F. (2020). Recruitment and Retention of Older People in Clinical Research: A Systematic Literature Review. J. Am. Geriatr. Soc..

[43] Prusaczyk B., Cherney S.M., Carpenter C.R., DuBois J.M. (2017). Informed Consent to Research with Cognitively Impaired Adults: Transdisciplinary Challenges and Opportunities. Clin. Gerontol..

[44] Mahmood A., Huang C.-M. (2024). From Our Lab to Their Homes: Learnings from Longitudinal Field Research with Older Adults. arXiv.

[45] Nunez M., Patel P., Ulin L., Kian L., Cominsky M., Burnett J., Lee J.L. (2025). Feasibility and Usage of a Virtual Assistant Device in Cognitively Impaired Homebound Older Adults. J. Appl. Gerontol..

[46] Mori M., MacDorman K., Kageki N. (2012). The Uncanny Valley [From the Field]. IEEE Robot. Autom. Mag..

[47] Busch P.A., Hausvik G.I., Ropstad O.K., Pettersen D. (2021). Smartphone Usage among Older Adults. Comput. Hum. Behav..

[48] Dino M.J.S., Dion K.W., Abadir P.M., Budhathoki C., Huang C.-M., Padula W.V., Himmelfarb C.R.D., Davidson P.M. (2024). The Impact of a Mixed Reality Technology-Driven Health Enhancing Physical Activity Program among Community-Dwelling Older Adults: A Study Protocol. Front. Public Health.

[49] Azeem S., Naveed M.S., Sajid M., Ali I. (2025). AI vs. Human Programmers: Complexity and Performance in Code Generation. VAWKUM Trans. Comput. Sci..

[50] Lukkahatai N., Joseph Dino M.N., Saligan L. (2025). Empowering Care: Transforming Nursing Through Artificial Intelligence. Artificial Intelligence in Medicine and Surgery—An Exploration of Current Trends, Potential Opportunities, and Evolving Threats.

[51] Dino M.J., Thiamwong L., Xie R., Malacas M.K., Hernandez R., Balbin P.T., Vital J.C., Rivero J.A., Xi V.W. (2025). Mobile Health (mHealth) Technologies for Fall Prevention among Older Adults in Low-Middle Income Countries: Bibliometrics, Network Analysis and Integrative Review. Front. Digit. Health.

[52] Cai T., Ma S., Zhong R. (2025). Can Intelligent Virtual Assistants Improve Cognitive Function in Older Adults? A Two-Wave Mediation Study. Digit. Health.

[53] Dino M.J.S., Dion K.W., Abadir P.M., Budhathoki C., Huang C.-M., Vital J.C., Rivero J.A., Malacas M.K., Hernandez R., Balbin P.T. (2025). Virtual Humans in Geriatric Care: An Integrative Review. J. Gerontol. Ser. A Biol. Sci. Med. Sci..

[54] Chaturvedi R., Verma S., Das R., Dwivedi Y.K. (2023). Social Companionship with Artificial Intelligence: Recent Trends and Future Avenues. Technol. Forecast. Soc. Chang..

