# Supplementary material for: Remote Virtual Interactive Agents for Older Adults: Exploring Its Science via Network Analysis and Systematic Review
Source: Healthcare (Basel). 2025 Sep 8;13(17):2253. doi: 10.3390/healthcare13172253 (PMC12427669; doi:10.3390/healthcare13172253)
Supplement: Supplementary file 1 [file healthcare-13-02253-s001.zip › healthcare-3844550-supplementary.pdf]

Supplementary File:

Table S1. Health Dimensions and Technology Characteristics of Virtual Interactive Agents for Older Adults.

No.	Authors	Purpose	Health	Agent	Device	Smart Capability	Connectivity
1	Bott et al. (2019)	Treatment/ Management	Multiple (Social and Emotional)	Visual - Non-humanoid (animal)	Tablet (App)	Non-AI (Programmed)	Online (Connected)
2	Bickmore et al. (2013)	Promotion (Normal)	Physical	Visual - Humanoid (avatar+)	Tablet (App)	Non-AI (Programmed)	Offline (Standalone)
3	Martinho et al. (2023)	Promotion (Normal)	Physical	Visual - Humanoid (avatar+)	Smartphone (App)	Non-AI (Programmed)	Online (Connected)
4	Hurmuz et al. (2022)	Promotion (Normal)	Multiple (Physical, Social, Cognitive)	Visual - Humanoid (avatar+)	Smartphone (App)	Non-AI (Programmed)	Online (Connected)
5	Kim & Kim (2024)	Promotion (Normal)	Multiple (Cognitive and Emotional)	Audio (Chatbot) only	Smartphone (App)	AI (Independent)	Online (Connected)
6	Jegundo et al. (2020)	Promotion (Normal)	Multiple	Visual - Humanoid (avatar+)	Multiple	AI (Independent)	Online (Connected)
7	Chi et al. (2016)	Prevention (High Risk)	Social	Visual - Humanoid (avatar+)	Multiple	Non-AI (Programmed)	Online (Connected)
8	Konig et al. (2017)	ADLs	Cognitive	Visual - Humanoid (avatar+)	Multiple	Non-AI (Programmed)	NA/NM
9	Ring et al. (2014)	Promotion (Normal)	Multiple	Visual - Humanoid (avatar+)	Computer (Software)	Non-AI (Programmed)	Offline (Standalone)
10	Bennion et al. (2020)	Promotion (Normal)	Multiple	Textual (Chatbot) only	Computer (Software)	Non-AI (Programmed)	Online (Connected)
11	Kramer et al. (2022)	Promotion (Normal)	Multiple	Visual - Humanoid (avatar+)	Multiple	AI (Independent)	Online (Connected)
12	Chou et al. (2024)	Multiple	Multiple	Textual (Chatbot) only	Smartphone (App)	Non-AI (Programmed)	Online
13	Azevedo et al. (2018)	Treatment/ Management	Cognitive	Visual - Humanoid (avatar+)	Multiple	NA/NM	Online
14	Takemoto et al. (2025)	Multiple	Multiple	Visual - Humanoid (avatar+)	Computer (Software)	AI (Independent)	Offline
15	Tokunaga et al. (2017)	ADLs	Multiple	Visual - Humanoid (avatar+)	Computer (Software)	AI (Independent)	Online
